# Effect of dexmedetomidine on cardiorespiratory regulation in spontaneously breathing adult rats

**DOI:** 10.1371/journal.pone.0262263

**Published:** 2022-01-14

**Authors:** Yoichiro Kitajima, Nana Sato Hashizume, Chikako Saiki, Ryoji Ide, Toshio Imai

**Affiliations:** Department of Physiology, The Nippon Dental University School of Life Dentistry at Tokyo, Tokyo, Japan; City of Hope National Medical Center, UNITED STATES

## Abstract

**Purpose:**

We examined the cardiorespiratory effect of dexmedetomidine, an α_2_- adrenoceptor/imidazoline 1 (I_1_) receptor agonist, in spontaneously breathing adult rats.

**Methods:**

Male rats (226−301 g, n = 49) under isoflurane anesthesia had their tail vein cannulated for drug administration and their tail artery cannulated for analysis of mean arterial pressure (MAP), pulse rate (PR), and arterial blood gases (PaO_2_, PaCO_2_, pH). After recovery, one set of rats received normal saline for control recording and was then divided into three experimental groups, two receiving dexmedetomidine (5 or 50 μg·kg^−1^) and one receiving normal saline (n = 7 per group). Another set of rats was divided into four groups receiving dexmedetomidine (50 μg·kg^−1^) followed 5 min later by 0.5 or 1 mg∙kg^−1^ atipamezole (selective α_2_-adrenoceptor antagonist) or efaroxan (α_2_-adrenoceptor/I_1_ receptor antagonist) (n = 6 or 8 per group). Recordings were performed 15 min after normal saline or dexmedetomidine administration.

**Results:**

Compared with normal saline, dexmedetomidine (5 and 50 μg·kg^−1^) decreased respiratory frequency (*f*_R_, p = 0.04 and < 0.01, respectively), PR (both p < 0.01), and PaO_2_ (p = 0.04 and < 0.01), and increased tidal volume (both p = 0.049). Dexmedetomidine at 5 μg·kg^−1^ did not significantly change minute ventilation (*V′*_E_) (p = 0.87) or MAP (p = 0.24), whereas dexmedetomidine at 50 μg·kg^−1^ significantly decreased *V′*_E_ (p = 0.03) and increased MAP (p < 0.01). Only dexmedetomidine at 50 μg·kg^−1^ increased PaCO_2_ (p < 0.01). Dexmedetomidine (5 and 50 μg·kg^−1^) significantly increased blood glucose (p < 0.01), and dexmedetomidine at 50 μg·kg^−1^ increased hemoglobin (p = 0.04). Supplemental atipamezole or efaroxan administration similarly prevented the 50 μg·kg^−1^ dexmedetomidine-related cardiorespiratory changes.

**Principal conclusion:**

These results suggest that dexmedetomidine-related hypoventilation and hypertension are observed simultaneously and occur predominantly through activation of α_2_-adrenoceptors, but not I_1_ receptors, in spontaneously breathing adult rats.

## Introduction

Dexmedetomidine provides its sedative and analgesic effects through its stimulant effect on α_2_-adrenoceptors. Its use, which was originally restricted to adult patients in intensive care units during mechanical ventilation [1, 2], has expanded, for example, to patients undergoing surgical operations (e.g. dental therapy) [3], or to infants and children undergoing nuclear medicine imaging examination [4]. In the clinical setting, it has been indicated that dexmedetomidine preserves ventilation and may be useful in patients with COVID-19 [5], but hypertension, hypotension and bradycardia are major complications limiting its use [2].

Dexmedetomidine is an α_2_-adrenoceptor/imidazoline 1 (I_1_) receptor agonist, and it has been suggested that activation of I_1_ receptors [6], as well as activation of α_2_-adrenoceptors [2], inhibits sympathetic outflow in the central nervous system and causes hypotension and bradycardia. However, there has been little attention paid to dexmedetomidine-related decrease in minute ventilation (*V′*_E_) [7, 8], because dexmedetomidine-related hypotension stimulates ventilation by activating chemoreceptors [9] and can minimize dexmedetomidine-related respiratory suppression.

Recently, we examined the cardiorespiratory effects of intraperitoneal injection of dexmedetomidine (50 μg·kg^−1^) in spontaneously breathing newborn rats (2−5 days old) [10, 11]. Our findings suggested that, in newborns, dexmedetomidine suppresses respiratory frequency and heart rate predominantly through α_2_-adrenoceptor activation [10, 11], whereas mean inspiratory flow (V_T_/T_I_, where V_T_ is tidal volume and T_I_ is inspiratory time) was stimulated by I_1_ receptor activation [11]. Hence, to extend our knowledge of respiratory regulation during dexmedetomidine administration, we examined cardiorespiratory indices in spontaneously breathing adult rats (8 weeks old), including V_T_/T_I_, mean arterial blood pressure (MAP), and arterial blood gases (ABGs). We also examined whether activation of I_1_ receptors, together with activation of α_2_-adrenoceptors, is involved in dexmedetomidine-related respiratory suppression by using two different antagonists, i.e. atipamezole (selective α_2_-adrenoceptor antagonist) and efaroxan (α_2_-adrenoceptor/I_1_ receptor antagonist) [11].

## Materials and methods

The experimental protocol was reviewed and approved by the Animal Research Committee of the Nippon Dental University School of Life Dentistry at Tokyo, Japan (Protocol Approved Numbers: 18-02-1 and 19–14). The animals were treated in accordance with the *Guiding Principles for the Care and Use of Animals in the Field of Physiological Sciences* (The Physiological Society of Japan), and we complied with the ARRIVE guidelines (https://journals.plos.org/plosbiology/article?id=10.1371/journal.pbio.1000412).

All efforts were made to minimize animal suffering and the number of animals used.

### Animals

Adult male Wistar rats were obtained from CLEA Japan Inc. (Tokyo, Japan) and maintained in the Nippon Dental University’s animal center at 22°C−25°C under a 12−hour:12−hour dark:light cycle with ad libitum access to food and water. On the day of the experiment, male rats (8 weeks old, 226−301 g; n = 49) were randomly assigned to the experimental groups. In the current study we used only male rats to exclude possible effects of gender difference on ventilation [12] and hypotension [13]; we plan a follow-up study with female animals in future.

### Drugs

We used the α_2_-adrenoceptor/I_1_ receptor agonist dexmedetomidine hydrochloride (Precedex; Maruishi Pharmaceutical Co., Osaka, Japan), the α_2_-adrenoceptor antagonist atipamezole (Antisedan; Orion Co., Farmos Group Ltd., Espoo, Finland), and the α_2_-adrenoceptor/I_1_ receptor antagonist efaroxan hydrochloride (Sigma-Aldrich, St. Louis, MO, USA). Dexmedetomidine (at 100 μg·mL^−1^ in normal saline), and atipamezole and efaroxan (each at 2.5 mg·mL^−1^ in normal saline) were stored in a freezer (−20°C). In the experiments, a single dose the drugs (i.e. normal saline or 5 or 50 μg·kg^−1^ dexmedetomidine, and 0.5 or 1.0 mg·kg^−1^ atipamezole or efaroxan), was administered at a volume of 0.4−0.5 mL·kg^−1^ with a 250−μL syringe (Gastight Syringe; Hamilton Company, Reno, NV, USA).

### Surgical preparations

On the day of the experiment, the rats were and anesthetized with 1%−3% isoflurane (Forane Inhalant Liquid; Abbott Japan Co., Ltd., Tokyo, Japan). Surgical preparations were performed under a surgical microscope (SZ60; Olympus, Tokyo, Japan). Briefly, local anesthetic (Xylocaine 2%; Aspen Japan K.K., Tokyo, Japan) was applied subcutaneously, and two incisions (each 5−10 mm long) were made in the ventral surface at the proximal end of the animal’s tail. At each incision, a catheter (24G × 1″, Nipro, Tokyo, Japan, and PE-50, Intramedic, Becton Dickinson and Company, Sparks, MD, USA) was gently inserted: one into a tail vein to administer the drug intravenously and one into the tail artery to monitor the pulse rate (PR) and mean arterial pressure (MAP) and to sample arterial blood for analysis of ABGs (PaO_2_, PaCO_2_, and pH), hemoglobin, hematocrit, electrolytes (Na^+^, K^+^, and Ca^2+^), and glucose. Each catheter was filled with saline-heparin solution (100 U·mL^−1^, a total volume of 0.1 mL). The rat was then given aspoxicillin (Doyle; Sawai Pharmaceutical Co.,Ltd, Osaka, Japan) and an analgesic (flurbiprofen axetil, 0.25 mg) intravenously, the catheters were fixed, and the incision was closed with instant adhesive. No bleeding or blood reflux was observed during the preparations. After returning the rat to its cage, we monitored its behavior carefully. We observed that all animals recovered consciousness within 5 min and could access water and laboratory chow by themselves. About 3 hours later, we started the recording.

**Fig 1:**
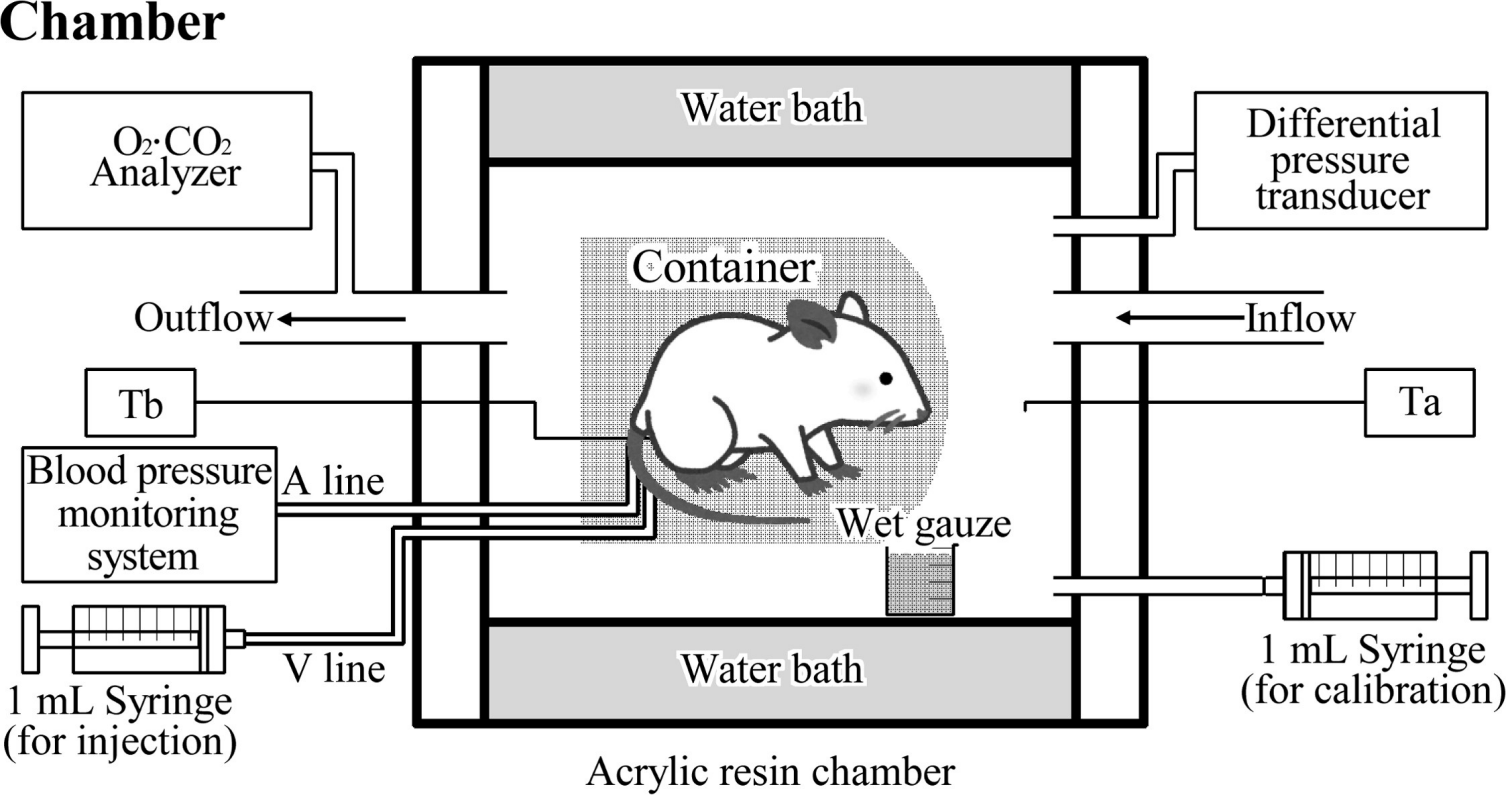
Chamber. Schematic diagram showing the experimental setup. O_2_·CO_2_ analyzer to measure the concentration of each gas (%) from the outflow; Differential pressure transducer to measure pressure changes in the chamber; A line, arterial catheter inserted into tail artery; V line, venous catheter inserted into tail vein; Ta and Tb, temperature probes to measure temperature inside the chamber (Ta) and the animal (Tb).

The animals were placed individually in a loose and flexible custom-made cylindrical container made of soft stainless-steel netting, in which the animal was able to roll and move back and forth. We used that container to avoid the risk of the animal turning back to bite or pull out the indwelling temperature probe or catheters during the measurements in the chamber (**Fig 1**). Chamber and body temperatures (°C) were monitored by means of fine chromel-alumel thermocouples (Omega Model 871a; Omega Engineering, Stamford, CT, USA). During the experiment, ambient temperature in the chamber was controlled at 25°C ± 2°C with the help of a circulating water bath (NCB-2510B; Tokyo Rikakikai Co. Ltd, Tokyo, Japan). To measure body temperature, the probe was inserted about 5 mm into the rectum with the aid of lidocaine jelly (Xylocaine Jelly; AstraZeneca K.K., Osaka, Japan) and lightly attached to the tail with a Band-Aid (Johnson & Johnson Services, Inc., New Brunswick, NJ, USA).

### Measurement of ventilation

Ventilation was measured by using a barometric technique [14, 15]. Briefly, we put the rat in the container into a cylindrical acrylic resin chamber (2300 mL) (**Fig 1**), and continuously delivered air through the chamber from the front (i.e. the inlet) to the back (i.e. the outlet) at a steady flow of 1400 mL·min^−1^ at STPD (standard temperature and pressure, dry) controlled by an adjustable flowmeter. To prevent the animal getting wet from urination during the recording, we laid a thick paper towel (Kim Towel, Nippon Paper Crecia Co., Ltd., Tokyo, Japan) beneath the container. Gas concentrations were monitored with a calibrated polarographic O_2_ analyzer and an infrared CO_2_ analyzer (Fox Box; Sable Systems International, North Las Vegas, NV, USA). To measure spontaneous breathing, the chamber inlet and outlet were temporarily closed, and the pressure oscillations in the recording chamber were monitored with a differential pressure transducer (DP45 ± 5.6 cm H_2_O; Validyne Engineering, Northridge, CA, USA) connected to a pre-amplifier (Model 1253A; San-ei Instruments, Tokyo Japan); these readings were displayed on a computer screen and recorded. The chamber was sealed for less than 1 min (mean 30 s), and, when it was reopened, the CO_2_ concentration at the outflow did not exceed 1%. We analyzed 20 to 50 regular breaths (mean 41 breaths), excluding spontaneous augmented breaths, to determine respiratory frequency (*f*_R_) and tidal volume (V_T_), from which we calculated minute ventilation (*V′*_E_ = *f*_R_·V_T_), total respiratory duration (T_TOT_), and inspiratory and expiratory time (T_I_ and T_E_). The volume was computed at BTPS (body temperature and pressure, saturated) and normalized by the weight of the animal in kilograms. The signal was calibrated for volume by injecting a known amount of air (e.g. 0.5 mL) when blood sampling was terminated at the end of the measurement.

### Measurement of pulse rate and mean arterial pressure

The arterial catheter was connected via a three-way stopcock to a liquid-filled pressure transducer (MLT0699; AD Instruments, Bella Vista, Australia) for the measurement of PR and MAP. The zero signal to the transducer was set to correspond to the chest level of the animal. The output was amplified (Model 2238; San-ei Instruments, Tokyo, Japan) and the signal was displayed on a computer screen and recorded.

### Data storage and analysis

The signals of ventilation, MAP, PR, temperatures, and in-and out-flowing gas concentrations (O_2_ and CO_2_) were monitored simultaneously and stored on a personal computer at a sampling frequency of 1 kHz for subsequent data analysis (PowerLab 4/25 and LabChart 8.0; AD Instruments).

### Arterial blood analysis (ABGs, electrolytes, glucose, hemoglobin, and hematocrit)

By using the arterial catheter, a 0.2 mL blood sample was collected anaerobically and immediately transferred into a disposable cartridge (CG8^+^; Abbot Japan) designed for the automated blood analyzer (i-STAT; Abbot Japan), Blood electrolytes (Na^+^, K^+^, Ca^2+^ [mmol·L^−1^]), blood glucose (mg·dL^−1^), hemoglobin (g·dL^−1^), and hematocrit (%), as well as ABGs (PaO_2_ and PaCO_2_ [mmHg]; pH), were measured by the automated blood analyzer. The values for ABGs were corrected with respect to body temperature, as proposed by Severinghaus [16]. Any blood remaining after the analysis was then returned to the animal, with a small amount of normal saline added to it to minimize loss in blood mass. In all groups, at each recording, the blood sampling was performed at the end (i.e. approximately 20 min after the administration of normal saline or dexmedetomidine).

### Protocols

The protocols used were based on those of our previous studies on newborn rats [10, 11]. Each intravenous administration (of normal saline, dexmedetomidine, atipamezole, or efaroxan), which was followed by flushing with normal saline (0.2 mL, corresponding to the liquid capacity of the venous catheter), was performed gently over a period of 1 min.

**Fig 2:**
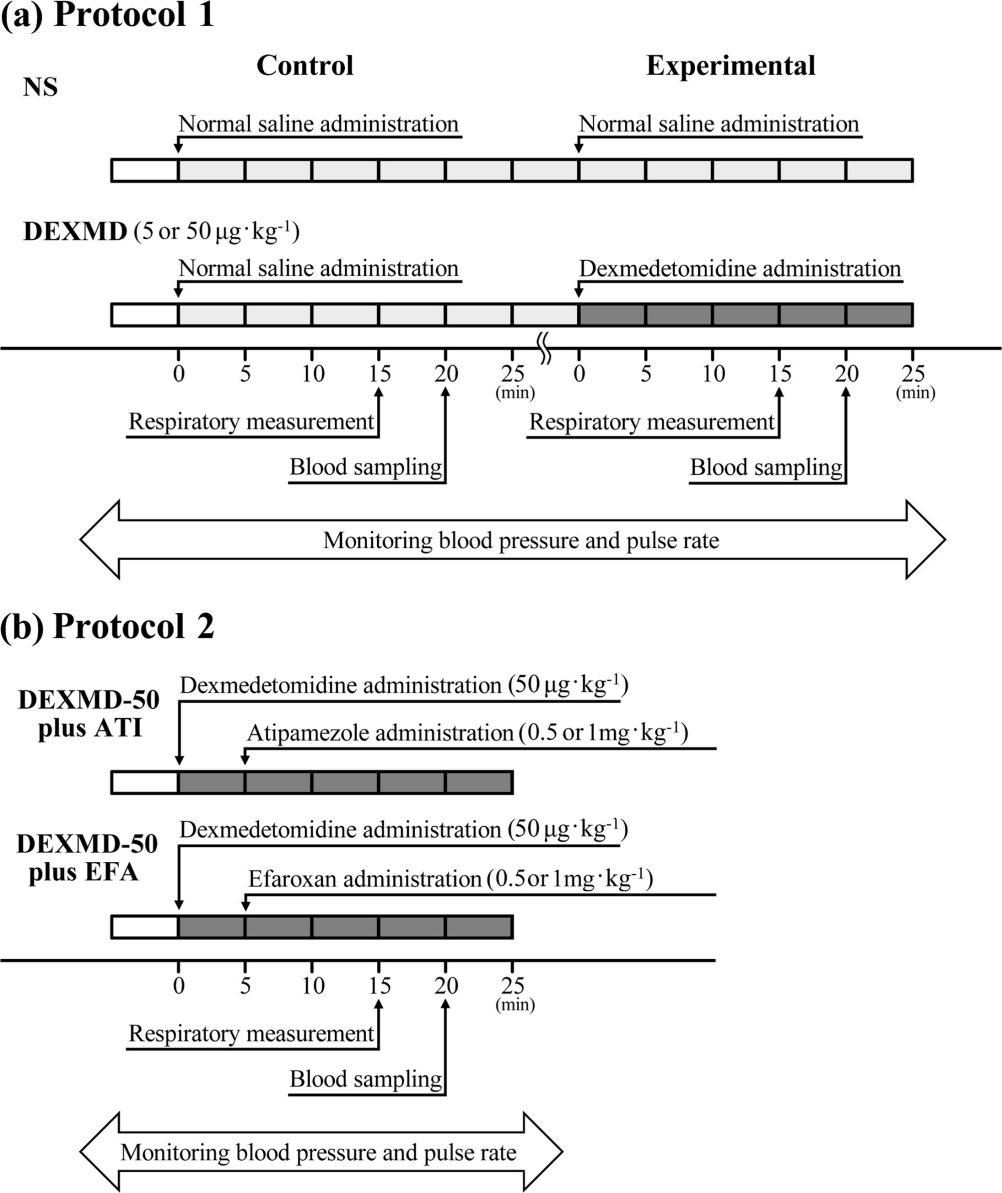
Protocols. **(a) Protocol 1:** Three groups of animals were prepared: NS, DEXMD-5, and DEXMD-50 (n = 7 in each group). All groups received only normal saline for control recording. At the experimental recording, animals received normal saline (NS) or dexmedetomidine (5 or 50 μg·kg^−1^) (DEXMD-5 and -50). Recordings were performed at 15 and 20 min after intravenous administration of normal saline or dexmedetomidine. **(b) Protocol 2:** Four groups of animals were prepared: DEXMD-50+ATI-0.5, DEXMD50+EFA-0.5, DEXMD-50+ATI-1.0, and DEXMD-50+EFA-1.0. They were respectively administered dexmedetomidine (50 μg·kg^−1^) followed 5 min later by 0.5 mg·kg^−1^ atipamezole (n = 8), 0.5 mg·kg^−1^ efaroxan (n = 8), 1.0 mg·kg^−1^ atipamezole (n = 6), or 1.0 mg·kg^−1^ efaroxan (n = 6), respectively. Recordings were performed at 15 and 20 min after intravenous administration of dexmedetomidine.

**Protocol 1:** After recovery from anesthesia in the recording chamber, a set of rats (249−300 g, n = 21) received normal saline for control recording and was then randomly divided into three groups to receive normal saline (NS), dexmedetomidine (5 μg·kg^−1^) (DEXMD-5), or dexmedetomidine (50 μg·kg^−1^) (DEXMD-50) (n = 7 in each group) for experimental recording **(Fig 2A)**.

**Protocol 2:** After recovery from anesthesia in the recording chamber, a set of rats (226−297 g, n = 28) was randomly divided into four groups to receive 50 μg·kg^−1^ of dexmedetomidine followed 5 min later by 0.5 mg·kg^−1^ of atipamezole or efaroxan (DEXMD-50+ATI-0.5 or DEXMD-50+EFA-0.5; n = 8 in each group), or 1.0 mg·kg^−1^ of atipamezole or efaroxan (DEXMD-50+ATI-1.0 or DEXMD-50+EFA-1.0) (n = 6 in each group) for experimental recording (**Fig 2B**). Basically, 0.5 mg·kg^−1^ of atipamezole [17] or efaroxan [18] was selected to prevent the effect of 50 μg·kg^−1^ of dexmedetomidine in rats, *in vivo* or *in vitro*, and further examination at a higher dose (i.e. 1.0 mg·kg^−1^) [10, 11] was added for each drug in this study. In Protocol 2, control recording (i.e. normal saline administration) was skipped, because intravenous volume loading could be excessive compared with that in Protocol 1. The summed data (n = 21) obtained at control recording in Protocol 1 were used as the data for NS in Protocol 2.

### Statistical analysis

Values are expressed as means and SD. The sample size for each cardiorespiratory index was determined by power analysis (α = 0.05 and β = 0.20) based on our previous study on newborn rats [11]; it was estimated that 6 to 7 animals per group were required.

For Protocol 1, the significance of differences among the three groups (NS, DEXMD-5, and DEXMD-50) was evaluated by one-way analysis of variance (ANOVA), and differences between groups in ANOVA were evaluated by Turkey-Kramer test. For Protocol 2, the summed data (n = 21) obtained at control recording in Protocol 1 were used as the data for the NS. The significance of differences among the three groups (NS, DEXMD-50+ATI-0.5, and DEXMD-50+EFA-0.5; or NS, DEXMD-50+ATI-1.0, and DEXMD-50+EFA-1.0) was evaluated by one-way analysis of variance (ANOVA), and differences between groups in the ANOVA were evaluated by Turkey-Kramer test. A p-value of less than 0.05 was considered significant.

Statistical analyses were performed by using BellCurve for Excel (Social Survey Research Information Co., Ltd., Tokyo, Japan).

## Results

### Protocol 1

The absolute values obtained at control recording are summarized in **Table 1**. Normal saline administration did not result in any difference among the groups, and we considered that all animals (n = 7 + 7 + 7 = 21) were basically from the same population.

**Table 1:** Absolute values at control recording (Protocol 1).

	NS (n = 7)		DEXMD-5 (n = 7)		DEXMD-50 (n = 7)	
	mean	SD	mean	SD	mean	SD
**Respiratory indices**						
***f***_**R**_ (breaths·min^−1^)	125	8	118	9	124	20
**V**_**T**_ (mL·kg^−1^)	6.00	1.03	6.50	0.93	5.95	1.06
***V′***_**E**_ (mL·min^−1^·kg^−1^)	749	121	744	93	732	124
**T**_**TOT**_ (s)	0.48	0.03	0.49	0.08	0.49	0.06
**T**_**I**_ (s)	0.19	0.01	0.20	0.02	0.19	0.02
**T**_**E**_ (s)	0.29	0.03	0.33	0.04	0.31	0.03
**V**_**T**_**/T**_**I**_ (mL·s^−1^·kg^−1^)	31.2	5.5	33.6	5.5	31.1	5.7
**T**_**I**_**/T**_**TOT**_	0.41	0.03	0.38	0.03	0.40	0.02
**Circulatory indices**						
**PR** (beats·min^−1^)	378	28	391	35	376	31
**MAP** (mmHg)	135	7	140	19	140	16
**Arterial blood data**						
**PaO**_**2**_ (mmHg)	95	4	91	4	92	3
**PaCO**_**2**_ (mmHg)	38.2	4.4	40.1	2.9	41.3	3.4
**pH**	7.40	0.02	7.43	0.03	7.42	0.01
**Ca**^**2+**^ (mmol·L^−1^)	1.27	0.09	1.33	0.07	1.35	0.08
**Na**^**+**^ (mmol·L^−1^)	147	4	144	3	143	2
**K**^**+**^ (mmol·L^−1^)	3.21	0.4	3.45	0.33	3.50	0.11
**Hemoglobin** (g·dL^−1^)	11.4	1.8	11.5	0.9	12.7	1.2
**Hematocrit** (%)	33	5	34	3	37	3
**Glucose** (mg·dL^−1^)	134	24	153	19	144	14
**Body temperature** (°C)	38.1	0.3	38.0	0.4	38.3	0.4

Values were obtained 15 and 20 min after a single dose administration of normal saline (vehicle, control) in three animal groups: NS, DEXMD-5, and DEXMD-50. At the control recording, all groups received only normal saline. There was no significant difference among the three groups at control recording. Hence, we considered the animals (in total, 7 + 7 + 7 = 21) to have been randomly selected from a single population.

**Fig 3:**
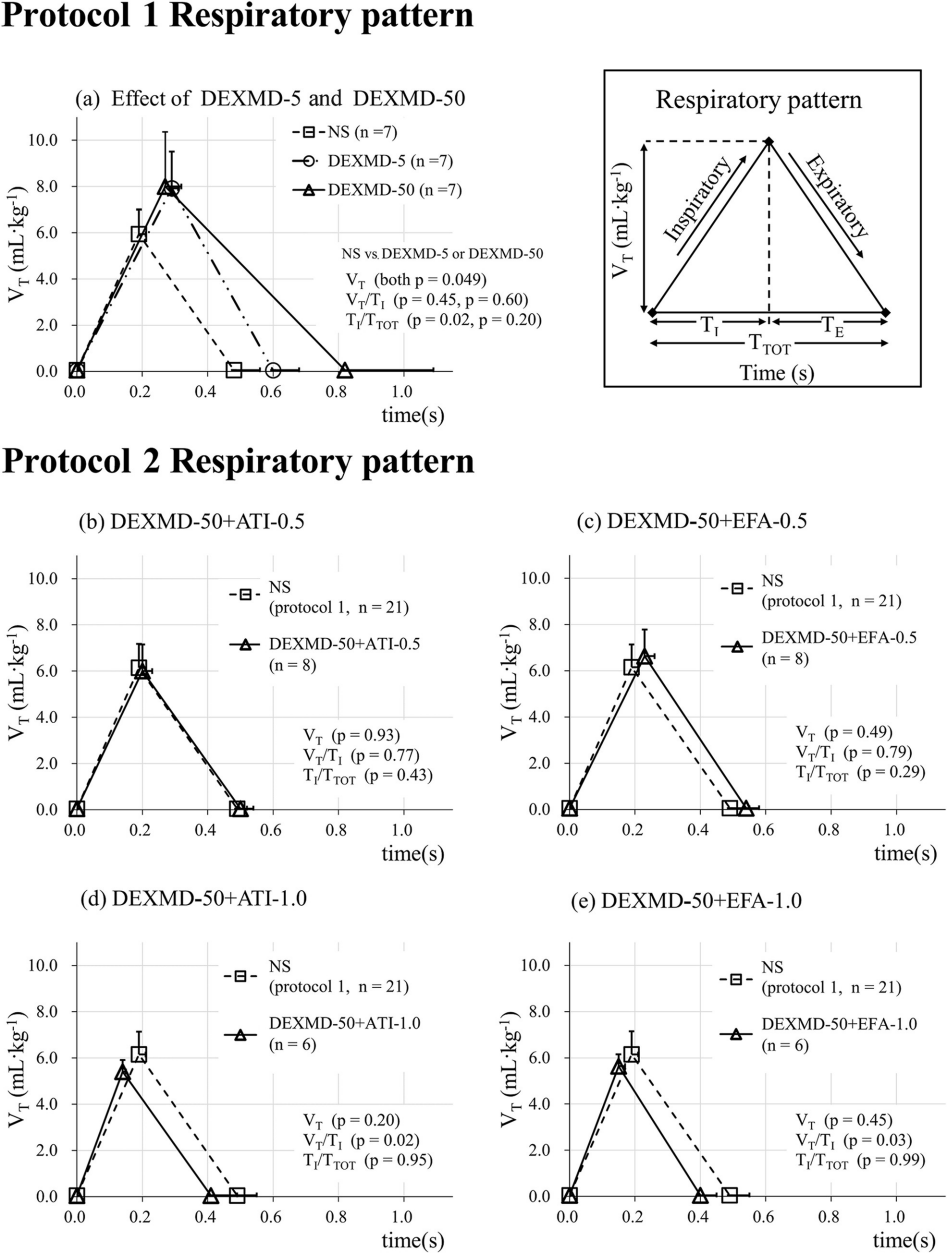
Respiratory pattern. **Protocol 1:** (a) Respiratory pattern in NS, DEXMD-5, and DEXMD-50, in which the rats were, respectively, given normal saline, dexmedetomidine (5 μg·kg^−1^), and dexmedetomidine (50 μg·kg^−1^), at experimental recording (n = 7 in each group). **Protocol 2:** (b−e) Respiratory patterns in DEXMD-50+ATI-0.5 (n = 8), DEXMD-50+EFA-0.5 (n = 8), DEXMD-50+ATI-1.0 (n = 6), or DEXMD-50+EFA-1.0 (n = 6), in which the rats were, respectively given dexmedetomidine (50 μg·kg^−1^), followed 5 min later by (b) atipamezole (0.5 mg·kg^−1^), or (c) efaroxan (0.5 mg·kg^−1^), or (d) atipamezole (1.0 mg·kg^−1^), or (e) efaroxan (1.0 mg·kg^−1^). In Protocol 2, data for the NS are the summed data (n = 21) obtained at control recording in Protocol 1.

**Table 2** summarizes the absolute values obtained at experimental recording. Compared with the NS, the DEXMD-5 and DEXMD-50 had decreased cardiorespiratory frequencies, i.e. *f*_R_ and PR, (*f*_R_, p = 0.04 and < 0.01, respectively; PR, both p < 0.01), and increased V_T_ (both p = 0.049); *V′*_E_ of DEXMD-5 was not significantly different (p = 0.87), whereas that of DEXMD-50 was significantly decreased (p = 0.03). In the analysis of breathing pattern, T_TOT_ in DEXMD-5 was not significantly different (p = 0.34) owing to significant T_I_ prolongation (p < 0.01) without T_E_ prolongation (p = 0.94), and T_TOT_ in DEXMD-50 was significantly prolonged owing to significant T_I_ prolongation with T_E_ prolongation (each p < 0.01). V_T_/T_I_ of the DEXMD-5 and DEXMD-50 was not significantly different (p = 0.45 and 0.60, respectively); T_I_/T_TOT_ of DEXMD-5 was significantly higher (p = 0.02), whereas that of DEXMD-50 was not significantly different (p = 0.20) (**Fig 3A**). In the analysis of circulation, MAP did not decrease in DEXMD-5 (p = 0.24), whereas it increased significantly in DEXMD-50 (p < 0.01). PR was significantly decreased in both DEXMD-5 and DEXMD-50 (both p < 0.01). Analysis of ABGs revealed that, although PaO_2_ decreased in both DEXND-5 and DEXMD-50 (p = 0.04 and p < 0.01, respectively), PaCO_2_ increased (p < 0.01) and pH decreased (p = 0.01) only in DEXMD-50. Arterial blood glucose increased in both DEXMD-5 and DEXMD-50 (both p < 0.01), and hemoglobin increased in DEXMD-50 (p = 0.04). In both DEXMD-5 and DEXMD-50, no significant change was observed in electrolytes, hematocrit, or body temperature.

**Table 2:** Effect of dexmedetomidine (5 μg∙kg^−1^ or 50 μg∙kg^−1^) at experimental recording (Protocol 1).

	NS (n = 7)		DEXMD-5 (n = 7)			DEXMD-50 (n = 7)			
	mean	SD	mean	SD	p values (vs. NS)	mean	SD	p values (vs. NS)	p values (DEXMD-5 vs. DEXMD-50)
**Respiratory indices**									
***f***_**R**_ (breaths·min^−1^)	126	9	102	16	* **0.04**	78	23	* **< 0.01**	^†^ **0.04**
**V**_**T**_ (mL·kg^−1^)	5.94	0.88	7.92	1.59	* **0.049**	8.00	2.36	* **0.049**	0.99
***V′***_**E**_ (mL·min^−1^·kg^−1^)	750	123	777	106	0.87	587	86	* **0.03**	^†^ **0.01**
**T**_**TOT**_ (s)	0.48	0.03	0.60	0.08	0.34	0.82	0.27	* **< 0.01**	0.06
**T**_**I**_ (s)	0.19	0.03	0.29	0.04	* **< 0.01**	0.27	0.05	* **< 0.01**	0.76
**T**_**E**_ (s)	0.29	0.01	0.31	0.08	0.94	0.55	0.23	* **< 0.01**	^†^ **0.02**
**V**_**T**_**/T**_**I**_ (mL·s^−1^·kg^−1^)	33.4	9.0	28.3	7.2	0.45	29.3	7.0	0.60	0.96
**T**_**I**_**/T**_**TOT**_	0.40	0.03	0.50	0.07	* **0.02**	0.35	0.07	0.20	^†^ **< 0.01**
**Circulatory indices**									
**PR** (beats·min^−1^)	384	42	324	25	* **< 0.01**	286	14	* **< 0.01**	0.06
**MAP** (mmHg)	137	13	121	21	0.24	182	14	* **< 0.01**	^†^ **< 0.01**
**Arterial blood data**									
**PaO**_**2**_ (mmHg)	91.6	5.5	83.0	6.0	* **0.04**	78.9	7.4	* **< 0.01**	0.55
**PaCO**_**2**_ (mmHg)	39.1	3.8	38.8	3.0	0.99	47.1	4.4	* **< 0.01**	^†^ **0.04**
**pH**	7.40	0.02	7.42	0.02	0.46	7.37	0.03	* **0.01**	^†^ **< 0.01**
**Ca**^**2+**^ (mmol·L^−1^)	1.29	0.12	1.35	0.03	0.30	1.37	0.06	0.12	0.89
**Na**^**+**^ (mmol·L^−1^)	146	3	143	2.5	0.054	147	2	0.09	0.96
**K**^**+**^ (mmol·L^−1^)	3.14	0.41	3.43	0.24	0.39	3.37	0.11	0.34	0.99
**Hemoglobin** (g·dL^−1^)	11.8	1.4	11.6	1.2	0.92	13.7	1.6	* **0.04**	^†^ **0.02**
**Hematocrit** (%)	35	4	34	3	0.95	40	5	0.17	0.11
**Glucose** (mg·dL^−1^)	143	14	249	26	* **< 0.01**	329	29	* **< 0.01**	^†^ **< 0.01**
**Body temperature** (°C)	38.1	0.5	37.8	0.8	0.78	37.8	0.9	0.99	0.75

*Significant difference (p < 0.05) between NS and DEXMD-5 or DEXMD-50.

^†^Significant difference (p < 0.05) between DEXMD-5 and DEXMD-50.

NS, DEXMD-5, and DEXMD-50 (n = 7 in each group) received normal saline, dexmedetomidine 5 μg·kg^−1^, and dexmedetomidine 50 μg·kg^−1^, respectively, at experimental recording.

Differences between DEXMD-5 and DEXMD-50 were significant in *f*_R_, *V′*_E_, T_E_, T_I_/T_TOT_, MAP, and arterial PaCO_2_, pH, hemoglobin and glucose.

### Protocol 2

The absolute values obtained in the DEXMD-50+ATI-0.5 or DEXMD-0.5+EFA-0.5 are summarized in **Table 3**, and the absolute values obtained in the DEXMD-50+ATI-1.0 and DEXMD+EFA-1.0 are summarized in **Table 4**. In both tables, the summed data obtained at control recording in Protocol 1 (n = 21) were used as the data for the NS.

**Table 3:** Effect of atipamezole or efaroxan (0.5 mg·kg^−1^) (Protocol 2).

	NS (n = 21)		DEXMD-50+ATI-0.5 (n = 8)			DEXMD-50+EFA-0.5 (n = 8)			
	mean	SD	mean	SD	p values (vs. NS)	mean	SD	p values (vs. NS)	p values (ATI-0.5 vs. EFA-0.5)
**Respiratory indices**									
***f***_**R**_ (breaths·min^−1^)	123	13	127	14	0.73	115	31	0.42	0.22
**V**_**T**_ (mL·kg^−1^)	6.15	0.99	6.00	1.15	0.93	6.63	0.93	0.49	0.43
***V′***_**E**_ (mL·min^−1^·kg^−1^)	742	108	728	179	0.96	746	86	0.99	0.95
**T**_**TOT**_ (s)	0.49	0.06	0.50	0.04	0.94	0.54	0.10	0.22	0.48
**T**_**I**_ (s)	0.19	0.02	0.20	0.03	0.67	0.23	0.05	* **0.03**	0.27
**T**_**E**_ (s)	0.31	0.04	0.29	0.04	0.74	0.28	0.10	0.97	0.70
**V**_**T**_**/T**_**I**_ (mL·s^−1^·kg^−1^)	32.0	5.4	30.3	7.9	0.77	30.4	4.8	0.79	0.99
**T**_**I**_**/T**_**TOT**_	0.40	0.03	0.42	0.05	0.43	0.42	0.05	0.29	0.97
**Circulatory indices**									
**PR** (beats·min^−1^)	384	31	309	15	* **< 0.01**	316	17	* **< 0.01**	0.80
**MAP** (mmHg)	138	14	143	20	0.44	131	15	0.25	0.36
**Arterial blood data**									
**PaO**_**2**_ (mmHg)	92.9	3.8	95.0	5.5	0.55	92.5	6.7	0.98	0.57
**PaCO**_**2**_ (mmHg)	37.8	9.6	39.4	8.5	0.89	35.6	6.8	0.83	0.67
**pH**	7.41	0.02	7.41	0.02	0.81	7.42	0.03	0.58	0.39
**Ca**^**2+**^ (mmol·L^−1^)	1.32	0.08	1.34	0.14	0.88	1.28	0.13	0.63	0.50
**Na**^**+**^ (mmol·L^−1^)	145	3	143	4	0.67	146	4	0.64	0.32
**K**^**+**^ (mmol·L^−1^)	3.4	0.3	3.4	0.6	0.99	3.2	0.5	0.53	0.60
**Hemoglobin** (g·dL^−1^)	11.9	1.4	12.3	2.0	0.80	10.9	1.8	0.35	0.22
**Hematocrit** (%)	35	4	36	6	0.75	32	5	0.38	0.21
**Glucose** (mg·dL^−1^)	143	21	201	31	* **< 0.01**	192	35	* **< 0.01**	0.80
**Body temperature** (°C)	38.1	0.4	38.1	0.5	0.78	38.1	0.4	0.77	0.99

*Significant difference (p < 0.05) between the NS and the DEXMD-50+ATI-0.5 or DEXMD-50+EFA-0.5.

No significant difference in any parameter was observed between DEXMD-50+ATI-0.5 and DEXMD-50+EFA-0.5.

Data for the NS are the summed data (n = 21) obtained at control recording in Protocol 1. Rats in the DEXMD-50+ATI-0.5 or DEXMD-50+EFA-0.5 (n = 8 in each group) were given dexmedetomidine (50 μg·kg^−1^) followed 5 min later by 0.5 mg·kg^−1^ atipamezole or efaroxan.

**Table 4:** Effect of atipamezole or efaroxan (1 mg·kg^−1^) (Protocol 2).

	NS (n = 21)		DEXMD-50+ATI-1.0 (n = 6)			DEXMD-50+EFA-1.0 (n = 6)			
	mean	SD	mean	SD	p values (vs. NS)	mean	SD	p values (vs. NS)	p values (ATI-1.0 vs. EFA-1.0)
**Respiratory indices**									
***f***_**R**_ (breaths·min^−1^)	123	13	160	41	* **< 0.01**	154	22	* **< 0.01**	0.85
**V**_**T**_ (mL·kg^−1^)	6.15	0.99	5.41	0.50	0.20	5.64	0.80	0.45	0.90
***V′***_**E**_ (mL·min^−1^·kg^−1^)	742	108	850	257	0.29	861	160	0.22	0.99
**T**_**TOT**_ (s)	0.49	0.06	0.41	0.08	* **0.01**	0.40	0.05	* **0.01**	0.96
**T**_**I**_ (s)	0.19	0.02	0.14	0.01	* **< 0.01**	0.15	0.02	* **< 0.01**	0.76
**T**_**E**_ (s)	0.31	0.04	0.27	0.08	0.24	0.25	0.04	* **0.04**	0.73
**V**_**T**_**/T**_**I**_ (mL·s^−1^·kg^−1^)	32.0	5.4	39.1	4.3	* **0.02**	38.6	6.5	* **0.03**	0.98
**T**_**I**_**/T**_**TOT**_	0.40	0.03	0.37	0.08	0.95	0.38	0.05	0.99	0.95
**Circulatory indices**									
**PR** (beats·min^−1^)	384	31	321	14	* **< 0.01**	317	37	* **< 0.01**	0.98
**MAP** (mmHg)	138	14	154	6	* **< 0.01**	156	14	* **< 0.01**	0.96
**Arterial blood data**									
**PaO**_**2**_ (mmHg)	92.9	3.8	96.7	9.0	0.29	94.3	5.6	0.82	0.74
**PaCO**_**2**_ (mmHg)	37.8	9.6	33.8	3.9	0.56	37.2	6.1	0.98	0.76
**pH**	7.41	0.02	7.40	0.01	0.17	7.42	0.01	0.94	0.20
**Ca**^**2+**^ (mmol·L^−1^)	1.32	0.08	1.25	0.05	0.19	1.29	0.11	0.78	0.66
**Na**^**+**^ (mmol·L^−1^)	145	3	147	5	0.49	145	2	0.98	0.72
**K**^**+**^ (mmol·L^−1^)	3.4	0.3	3.1	0.3	0.14	3.2	0.3	0.48	0.81
**Hemoglobin** (g·dL^−1^)	11.9	1.4	10.8	1.0	0.20	11.7	1.4	0.93	0.51
**Hematocrit** (%)	35	4	32	3	0.24	34	4	0.96	0.51
**Glucose** (mg·dL^−1^)	143	21	164	19	* **0.04**	193	30	* **< 0.01**	0.06
**Body temperature** (°C)	38.1	0.4	37.9	0.4	0.87	38.2	0.5	0.82	0.99

*Significant difference (p < 0.05) between the NS and the DEXMD-50+ATI-1.0 or the DEXMD-50+EFA-1.0.

No significant difference in any parameter was observed between DEXMD-50+ATI-1.0 and DEXMD-50+EFA-1.0.

Data for the NS are the summed data (n = 21) obtained at control recording in Protocol 1. Rats in the DEXMD-50+ATI-1.0 and DEXMD-50+EFA-1.0 (n = 6 in each group) were given dexmedetomidine (50 μg·kg^−1^), followed 5 min later by 1.0 mg·kg^−1^ atipamezole or efaroxan.

As shown in **Table 3**, most of the results in the DEXMD-50+ATI-0.5 and DEXMD-50+EFA-0.5 were not significantly different from those in the NS, with the exception of T_I_ (p = 0.03, only in DEXMD-50+EFA-0.5), PR (both p < 0.01), and arterial blood glucose (both p < 0.01) (**Table 3, Fig 3B and 3C**). No significant difference was observed between any parameter in the DEXMD-50+ATI-0.5 and the DEXMD-50+EFA-0.5.

As shown in **Table 4**, in DEXMD-50+ATI-1.0 and DEXMD-50+EFA-1.0, *f*_R_ (both p < 0.01) and V_T_/T_I_ (p = 0.02 and 0.03, respectively) were higher than in the NS owing to significantly shortened T_TOT_ (both, p = 0.01) and T_I_ (both p < 0.01), respectively (**Fig 3D and 3E**). In the analysis of circulation, MAP increased (both p < 0.01), whereas PR decreased (both p < 0.01), compared with those in the NS (**Table 4**). In addition, arterial blood glucose increased compared with that in the NS (p = 0.04 and < 0.01, respectively). No significant difference was observed between any parameter in the DEXMD-50+ATI-1.0 and the DEXMD-50+EFA-1.0.

**Fig 3** graphically shows the respiratory pattern of NS, DEXMD-5, and DEXMD-50 (Protocol 1) (**Fig 3A**) and those of the DEXMD-50+ATI-0.5, DEXMD-50+EFA-0.5, DEXMD-50+ATI-1.0, or DEXMD-50+EFA-1.0 (Protocol 2) (**Fig 3B–3E**). In Protocol 1, DEXMD-5 and DEXMD-50 prolonged T_I_ (both p < 0.01) compared with NS and increased V_T_ (both p = 0.049), and these significant changes resulted in there being no change in V_T_/T_I_ (p = 0.45 and p = 0.60, respectively), which is the slope at inspiration and an index of respiratory drive. In Protocol 2, in the DEXMD-50+ATI-0.5 (**Fig 3B**) or DEXMD-50+EFA-0.5 (**Fig 3C**), V_T_/T_I_ remained comparable to that of the NS (n = 21) (p = 0.77 and p = 0.79, respectively), without changes in V_T_ (p = 0.93 and p = 0.49, respectively) and T_I_/T_TOT_ (p = 0.43 and p = 0.29, respectively). In the DEXMD-50+ATI-1.0 (**Fig 3D**) and DEXMD-50+EFA-1.0 (**Fig 3E**), V_T_/T_I_ significantly increased (p = 0.02 and p = 0.03, respectively) without changes in V_T_ (p = 0.20 and 0.45, respectively) and T_I_/T_TOT_ (p = 0.95 and p = 0.99, respectively).

## Discussion

In previous studies, we examined the cardiorespiratory effects of an intraperitoneal injection of dexmedetomidine (50 μg·kg^−1^) on spontaneously breathing newborn rats [10, 11]. We found that the cardiorespiratory suppression that occurred following administration of dexmedetomidine was reversed by the addition of atipamezole (a selective α_2_-adrenoceptor antagonist) [10]. Similar dexmedetomidine-mediated changes in respiration-related activities have been seen in a newborn rat *in vitro* brainstem-spinal cord preparation [19], suggesting that dexmedetomidine affects the generation of respiratory rhythm at the level of the brainstem and spinal cord. Furthermore, from a comparison of the results of dexmedetomidine plus atipamezole with those of dexmedetomidine plus efaroxan (the latter being an α_2_-adrenoceptor/I_1_ receptor antagonist), we hypothesized that I_1_ receptor stimulation due to dexmedetomidine was a factor involved in maintaining V_T_/T_I_ (an index of respiratory drive) in newborn rats [11]. Although the stimulatory effect of dexmedetomidine on the I_1_ receptor is unlikely to be greater than its effect on the α_2_-adrenoceptor [20], our hypothesis seems consistent with clinical observations that suggest that dexmedetomidine does not severely suppress ventilation [1, 2, 4].

However, it is unknown whether this hypothesis on dexmedetomidine and I_1_ receptor activation can be applied to adult rats, in which, for example, the respiratory mechanics, breathing patterns, and lung volumes per bodyweight differ from those in newborn rats [21, 22] and may influence the values of V_T_/T_I_ [23]. Hence, to add information related to maturity, we examined spontaneously breathing adult rats in this study by following essentially the same protocol as that which we applied to newborn rats [10, 11]. The difference was that two different doses (5 and 50 μg·kg^−1^) were prepared (Protocol 1) in consideration of possible age-related differences in drug sensitivity [24] and in pharmacokinetics and pharmacodynamics [2], as well as of the possible effect of dexmedetomidine on MAP [25], which is unmeasurable in newborn rats [10, 11].

Compared with the mean values of NS (= 100%) (**Table 2**), administration of 5 μg·kg^−1^ of dexmedetomidine decreased *f*_R_ to approximately 81% and PR to approximately 84% (p = 0.04 and p < 0.01, respectively), and 50 μg·kg^−1^ of dexmedetomidine decreased *f*_R_ to 62% and PR to 74% (both p < 0.01). V_T_ was increased to 133% and 135% by administration of 5 and 50 μg·kg^−1^ dexmedetomidine, respectively (p = 0.049), but *V′*_E_ (the product of *f*_R_ and V_T_) was decreased (to 78%; p = 0.03) only by 50 μg·kg^−1^ dexmedetomidine. In taking this information together with the results obtained in previous studies of newborn rats given 50 μg·kg^−1^ of dexmedetomidine [10, 11], we found that administration of 50 μg·kg^−1^ of dexmedetomidine consistently resulted in more severe suppression of *f*_R_ relative to heart rate and increased V_T_, irrespective of whether the animal was mature or newborn. In addition, rats administered 5 or 50 μg·kg^−1^ dexmedetomidine showed hypoxemia (i.e. decrease in PaO_2_) (p = 0.04 and p < 0.01, respectively), hyperglycemia (i.e. increase in blood glucose) (both p < 0.01), and increase in hemoglobin (p = 0.92 and p = 0.04). In rats given 50 μg·kg^−1^ of dexmedetomidine, the significantly decreased *V′*_E_ (p = 0.03) was consistent with the results of ABGs, which indicated hypoventilation (i.e. increase in PaCO_2_) (p < 0.01) and acidemia (i.e. decrease in pH) (p = 0.01). MAP was not changed significantly by 5 μg·kg^−1^ of dexmedetomidine (p = 0.24) but was increased to 133% by 50 μg·kg^−1^ dexmedetomidine (p < 0.01). In adult male human volunteers, incrementally administered dexmedetomidine gradually increases MAP (+ 12% from baseline) after transient hypotension (−13%), and the increased MAP coincides with a drop in heart rate and stroke volume (and hence cardiac output), increases in pulmonary and systemic vascular resistance, and a slight increase in PaCO_2_ [25]. Hence, in our rats, it is possible that the increased PaCO_2_ (**Table 2**) was induced by suppression of both ventilation and cardiac output upon administration of 50 μg·kg^−1^ of dexmedetomidine. The results of Protocol 1 (**Tables 1 and 2**), suggest that the effects of 50 μg·kg^−1^ dexmedetomidine are not merely suppressive (e.g. on the *V′*_E_ and PR), but also stimulatory (e.g. on the V_T_, MAP, hemoglobin, and glucose), in spontaneously breathing adult rats.

In Protocol 2, the summed data (n = 21) obtained at control recording in Protocol 1 were used as the data for the NS. All animals were administered 50 μg·kg^−1^ of dexmedetomidine (**Fig 2B**). Compared with the mean values of NS (n = 21), administration of 0.5 mg·kg^−1^ atipamezole or efaroxan in addition to 50 μg·kg^−1^ of dexmedetomidine prevented changes in most of the cardiorespiratory indices affected by administration of 50 μg·kg^−1^ dexmedetomidine alone (**Table 3**). In earlier studies, almost complete prevention of the dexmedetomidine-related physiological changes was obtained when dexmedetomidine and atipamezole were used in a ratio of 1 to 10 (i.e. 100 μg·kg^−1^ dexmedetomidine and 1.0 mg∙kg^−1^ atipamezole) in fentanyl/nitrous oxide-anesthetized adult rats [17], or when dexmedetomidine and efaroxan were used in a ratio of 1 to 10 (i.e. 10^−6^ M dexmedetomidine and 10^−5^ M efaroxan) in adult rat hippocampal slices [18]. Similarly, we found no significant difference in any parameter, except PR and glucose (both p < 0.01) in rats administered 0.5 mg·kg^−1^ atipamezole in addition to 50 μg·kg^−1^ dexmedetomidine, and T_I_, PR, and blood glucose (p = 0.03, p < 0.01, p < 0.01, respectively) in rats administered 0.5 mg·kg^−1^ efaroxan in addition to 50 μg·kg^−1^ dexmedetomidine. Moreover, in this experiment, no significant difference in any parameter, including V_T_/T_I_ (**Fig 3B−3E**), was observed between rats given 0.5 mg·kg^−1^ of atipamezole and efaroxan (**Table 3**) or 1.0 mg·kg^−1^ of atipamezole and efaroxan (**Table 4**) in addition to 50 μg·kg^−1^ dexmedetomidine.

Dexmedetomidine activates both α_2_-adrenoceptors and I_1_ receptors, and, in theory, supplemental administration of atipamezole (a selective α_2_-adrenoceptor antagonist) would block only α_2_-adrenoceptor activation, whereas supplemental administration of efaroxan (an α_2_-adrenoceptor/I_1_ receptor antagonist) would block the activation of both α_2_-adrenoceptors and I_1_ receptors. Hence, the similarity in the effects of supplemental atipamezole and efaroxan administration suggests that dexmedetomidine-related cardiorespiratory changes in spontaneously breathing adult rats occur predominantly through α_2_-adrenoceptor activation, not I_1_ receptor activation.

In our previous study on spontaneously breathing newborn rats, V_T_/T_I_ was not affected by dexmedetomidine (50 μg·kg^−1^) alone or by dexmedetomidine (50 μg·kg^−1^) plus 1, 5, or 10 mg·kg^−1^ of atipamezole, but it was significantly decreased by dexmedetomidine (50 μg·kg^−1^) plus 1, 5, or 10 mg·kg^−1^ of efaroxan; therefore, we concluded that it is I_1_ receptor activation that maintains V_T_/T_I_, (i.e. an index of respiratory drive) in newborn rats [11]. In contrast, in the present study, the distinct effect of I_1_ receptor activation on V_T_/T_I_ was not apparent in spontaneously breathing adult rats (**Fig 3B−3E**). Together, these results on adult and newborn rats suggest that the functional roles of α_2_-adrenoceptors and I_1_ receptors on the cardiorespiratory system differ between immature and mature animals.

As limitations of the study, we cannot exclude the possible influences of isoflurane anesthesia and flurbiprofen axetil, which we administered for surgical preparation before the recordings. In addition, we did not directly measure the flow signals but instead used a barometric method to measure the fluctuations caused in chamber pressure by respiratory movement. Therefore, for example, V_T_ can be overestimated in cases where the respiratory flow resistance is high [26]. However, this seems unlikely, because an earlier study on adult rats under mechanical ventilation reported that dexmedetomidine (250 μg·kg^−1^ intraperitoneal injection followed by intravenous infusion of 0.5 μg·kg^−1^) did not significantly change respiratory mechanical parameters in comparison with those measured in animals that received diazepam (5 mg) and pentobarbital (20 mg·kg^−1^), intraperitoneally [7]. Ventilation is under the influence of circadian rhythm [27] and V_T_/T_I_ is reported to increase with age. In human infants, “on-switching” and “off-switching” of inspiratory activity may depend on the sleep state [28], and the lengths of time spent in different sleep states (i.e. rapid-eye-movement (REM) sleep (or active sleep) [29] and quiet sleep) change with postnatal development [30]. REM sleep can be suppressed by clonidine [31], which is another clinically used α_2_-adrenoceptor/I_1_ receptor agonist [1, 6]. In this study, although we restricted our measurements to the afternoon (approximately 14:00−16:00), it was not clear how dexmedetomidine administration affected sleep state of the spontaneously breathing animal during the measurements.

We observed significantly increased hemoglobin after administration of 50 μg·kg^−1^ dexmedetomidine (**Table 2**) and the effect was prevented by atipamezole or efaroxan (**Tables 3 and 4**); this might have due to dexmedetomidine-related diuretic [32] and hyperglycemic effects [33]. The hemoconcentration increases viscosity [34] and may obstruct circulatory O_2_ and CO_2_ transport and secondarily stimulate sympathetic outflow. However, unlike the effect on MAP, blockade of the α_2_-adrenoceptor or the α_2_-adrenoceptor/I_1_ receptor by atipamezole and efaroxan could not completely prevent the dexmedetomidine-related decrease in PR (**Tables 3 and 4**), and the results were similar to those observed previously in newborn rats [10, 11]. It is possible that PR is influenced by a reflex bradycardia, either with possible enhancement of the reflex bradycardia through the α_2_-adrenoceptor (i.e. by administration of dexmedetomidine alone) or without this enhancement (i.e. by administration of dexmedetomidine plus atipamezole or efaroxan) [35]. Further investigation of the mechanisms underlying the persistent drop in the PR is warranted. In other words, from a practical point of view, the results suggest that *f*_R_ is a better indicator than heart rate or PR for monitoring the cardiorespiratory effects of dexmedetomidine or its antagonists (e.g. atipamezole or efaroxan).

## Conclusions

The results of this study suggest that not all cardiorespiratory indices are suppressed by dexmedetomidine in spontaneously breathing adult rats: some (V_T_ and MAP) are stimulated to increase. The similarity in the effects of supplemental administration of atipamezole and efaroxan suggests that dexmedetomidine-related decrease in *V′*_E_ and increase in MAP are observed simultaneously and occur predominantly through activation of α_2_-adrenoceptors, but not I_1_ receptors, in the spontaneously breathing adult rats.
